# Scoping review of toolkits as a knowledge translation strategy in health

**DOI:** 10.1186/s12911-014-0121-7

**Published:** 2014-12-24

**Authors:** Raluca Barac, Sherry Stein, Beth Bruce, Melanie Barwick

**Affiliations:** Hospital for Sick Children, Toronto, Canada; Dalhousie University, Halifax, Canada; University of Toronto, Toronto, Canada

**Keywords:** Toolkit, Knowledge translation, Practice change, Evaluation, Health

## Abstract

**Background:**

Significant resources are invested in the production of research knowledge with the ultimate objective of integrating research evidence into practice. Toolkits are becoming increasingly popular as a knowledge translation (KT) strategy for disseminating health information, to build awareness, inform, and change public and healthcare provider behavior. Toolkits communicate messages aimed at improving health and changing practice to diverse audiences, including healthcare practitioners, patients, community and health organizations, and policy makers. This scoping review explores the use of toolkits in health and healthcare.

**Methods:**

Using Arksey and O’Malley’s scoping review framework, health-based toolkits were identified through a search of electronic databases and grey literature for relevant articles and toolkits published between 2004 and 2011. Two reviewers independently extracted data on toolkit topic, format, target audience, content, evidence underlying toolkit content, and evaluation of the toolkit as a KT strategy.

**Results:**

Among the 253 sources identified, 139 met initial inclusion criteria and 83 toolkits were included in the final sample. Fewer than half of the sources fully described the toolkit content and about 70% made some mention of the evidence underlying the content. Of 83 toolkits, only 31 (37%) had been evaluated at any level (27 toolkits were evaluated overall relative to their purpose or KT goal, and 4 toolkits evaluated the effectiveness of certain elements contained within them).

**Conclusions:**

Toolkits used to disseminate health knowledge or support practice change often do not specify the evidence base from which they draw, and their effectiveness as a knowledge translation strategy is rarely assessed. To truly inform health and healthcare, toolkits should include comprehensive descriptions of their content, be explicit regarding content that is evidence-based, and include an evaluation of the their effectiveness as a KT strategy, addressing both clinical and implementation outcomes.

## Background

Each year, considerable human and financial resources are devoted to the development of research knowledge in various sectors of health (e.g., clinical, biomedical, health services, population health). The ultimate objective on the research continuum is to integrate research evidence into practice to inform and improve health outcomes. To this end, efforts in the rapidly developing field of knowledge translation (KT) have focused on bridging research to practice. Knowledge translation efforts in health strive to ensure that knowledge users are aware of and use research evidence to inform health and healthcare decision-making [1]. In other words, knowledge users need to access research evidence and know what to do with the knowledge shared in order to impact health and clinical care, whether through increased awareness, sharing knowledge, or facilitating behavior/practice change.

### Effectiveness of KT strategies

A range of KT strategies have been used with varied effectiveness to increase awareness and utilization of research evidence in health, including but not limited to printed educational materials (e.g., guidelines for practice, audio-visual materials, electronic publications, and multifaceted combinations of these elements in the form of toolkits), educational meetings (e.g., conferences, workshop, lectures), educational outreach (or academic detailing, which supports change through one-on-one engagement between expert and practitioner), local opinion leaders (providers who are educationally influential), audit and feedback (any summary of clinical performance of healthcare to change behavior over a specified period of time), and reminders (patient or encounter specific prompts). KT strategies can also be tailored, taking into account identified barriers to change, and/or multifaceted, which capture interventions having two or more components [1]. Despite over 300 systematic reviews of professional behavior change conducted by Cochrane Effective Practice and Organisation of Care Group (EPOC), evidence on the effectiveness of different strategies remains incomplete [1].

Most of the reviews included in Bero et al.’s systematic review identified modest improvements in performance resulting from these common KT interventions [2]. Generally, consistently effective interventions include reminders, multifaceted interventions, and interactive educational meetings. KT strategies having variable effectiveness include audit and feedback, local opinion leaders, local consensus processes, and patient mediated interventions. KT strategies with little or no effect included educational materials and didactic education meetings. Multifaceted interventions appear to be more effective than single KT interventions [2,3], although others have shown that single KT strategies can be as effective at changing knowledge (but not practice) as multifaceted ones when they utilized tailored and targeted messaging [4].

The vision of mapping which KT strategies are effective for which audiences and in what contexts remains elusive. The field is in need of more detailed guidance on how to best share research evidence in a way that promotes and enables its use. Some of this guidance is emerging from taxonomies of KT strategies developed recently to facilitate greater consistency in strategy naming, definition, level of detail and specification of the target user in research on KT strategy effectiveness, with the ultimate goal of enhancing the synthesis and application of findings across a body of research [5,6]. Context appears to be a defining feature in KT for practice change, and it is highly likely that no single KT strategy will prove to be universally effective across contexts. Recent developments in the KT field have identified a range of contextual factors that are implicated in successful practice change [7] and this emerging evidence can shape how we explore the effectiveness of KT strategies to maximize their impact. On the practice side, organizational efforts to integrate research findings into practice are on the rise in a range of practice settings yet such efforts often do not include an evaluation component to identify whether the strategy met the intended KT goal. Indeed, both research and practice change endeavors rarely explore both clinical and implementation outcomes stemming from practice change initiatives, a situation that lends itself to a Type III error. Practice change initiatives need to evaluate the fidelity with which clinical interventions are implemented in order to accurately discern whether a failure to demonstrate intended clinical outcomes is due to poor implementation or weaknesses inherent in the clinical intervention itself [8].

### Educational meetings and materials

Educational meetings and materials tend to be the most widely used KT strategies in health and healthcare [4]; they are simple and well known, incur the least costs, and are feasible to use in many contexts. However, despite wide spread use, research on the effectiveness of educational materials and meetings is inconclusive. This may be due to differences in the characteristics of the educational materials, including their attractiveness, content, format, mode of delivery, timing, frequency, and complexity of targeted behaviour change or other KT goal [4]. A Cochrane systematic review on the use of educational materials to change the practice of healthcare professionals and patient health outcomes showed that 98% of the studies compared the use of educational materials to no intervention, making it difficult to ascertain the effectiveness of printed educational materials relative to other KT strategies [9]. Overall, printed educational materials were found to have a small positive effect on professional practice outcomes and an unknown effect on patient outcomes, as 91% of the studies did not report on patient outcomes. Although educational materials are widely used, we still lack primary research on their effectiveness, and how their content, format and delivery can be optimized to increase their effectiveness. Given that educational materials are, and will likely continue to be widely used to share knowledge and change practice, optimizing their use should be a priority in the health field.

### Toolkits

Educational materials are often packaged as “toolkits”. Coined in the 1980’s, the term ‘toolkit’ has been widely used for decades. As in other fields, no definitive toolkit format has been established in the context of health. The term has been used to describe the bundling of a combination of educational materials including templates, instruction sheets, literature reviews, videos, and posters, presented in a variety of formats (hard copy, web). Toolkits have been used to inform and improve health behaviors for diverse audiences, including health practitioners, patients, community and health organizations, policy makers, and for the public. Toolkits have gained popularity as a KT strategy, particularly in health, and the Internet has provided fertile ground for toolkit dissemination. For example, a recently completed study of evidence-based interventions to increase booster seat use lends itself to dissemination in a web-based toolkit format for multiple target audiences that are highly likely to access information on the Web [Barwick, Bruce, Fuselli, Stein, & Barac: Impact and sustainability of targeted booster seat activities among community organizations in injury prevention, submitted].

Given the pervasiveness of toolkits to disseminate health evidence and change practice, it is important to map the evidence for their effectiveness to inform decisions about their potential use. To this end, the present study focuses on toolkits as a KT strategy. We conducted a scoping review of published and grey literature to identify how toolkits have been operationalized, and to describe their content, evidence underlying their content, format, and evidence of effectiveness. Our focus was on toolkits that were developed to disseminate health information, as well as change healthcare provider behavior (i.e., both health- and healthcare-focused). As is the purview of scoping reviews, we sought to assess whether sufficient research is available to warrant a more traditional systematic review on health toolkits and, where gaps are noted, to issue recommendations for future research on toolkit development and effectiveness. This type of review is appropriate given the emerging nature of evidence on toolkits and the absence of syntheses on this topic [10].

## Methods

The review followed Arksey and O’Malley scoping review framework [11] and the PRISMA flow diagram for reporting standards in systematic reviews and meta-analyses [12]. Arskey and O’Malley’s framework includes five key phases: (i) identifying the research question; (ii) identifying relevant studies; (iii) study selection; (iv) charting the data; and (v) collating, summarizing and reporting the results. Ethics approval was exempt for the present study because no data collection was required.

### Definitions

While no single definition of the term toolkit has gained wide acceptance, for the purposes of this review ‘toolkit’ was defined as the packaging of multiple resources that codify explicit knowledge, such as templates, pocket cards, guidelines, algorithms, summaries, and that are geared to knowledge sharing, educate, and/or facilitate behavior change. For example, *How Schools Can Help Students Recover from Traumatic Experiences* is an online toolkit designed to support schools that want to help students recover from traumatic experiences such as natural disasters, exposure to violence, abuse or assault, terrorist incidents, war and refugee experiences [13]. The toolkit lists existing programs for each trauma type, providing program goal, program delivery and implementation requirements to facilitate the comparison of different programs available to schools having this goal.

### Search strategy

The review sought to assess the scope of toolkits available in the field of health and healthcare, so search terms were relatively broad. Electronic databases and websites were searched by a library and information scientist. The research team compiled a broad list of terms pertinent to health and healthcare. These terms were used by the library scientist in various combinations to search electronic databases of peer-reviewed and grey literature. Searches were limited to English language publications from 2004 to 2011. Papers were included if they reported on toolkits used for disseminating health information and informing and/or changing healthcare provider behavior. Given the goal of mapping how toolkits have been used as a KT strategy, study design was left unspecified. The following electronic databases were searched: MEDLINE, PsychINFO, CINAHL, ERIC and those available in Scholar’s Portal. Common terms across all databases included health education and health promotion. Each database contained unique relevant terms used to create search strings. The term ‘toolkit’ was included as a keyword as it is not used in the controlled vocabulary for these databases. The following headings were used in combination with ‘tool kit’ or ‘toolkit’ to create search strings: health education, health promotion, consumer health information, preventative health services, information resources, child safety, passenger safety, patient safety, health programs and educational programs.

Grey literature was searched using Scirus (now retired), a scientific search engine that searches journals as well as websites. The search terms included toolkit or tool kit, health, child, pediatric, youth. Searches were limited to the following subject areas: medicine, psychology, social and behavioral sciences and sociology.

### Source selection

**Figure 1:**
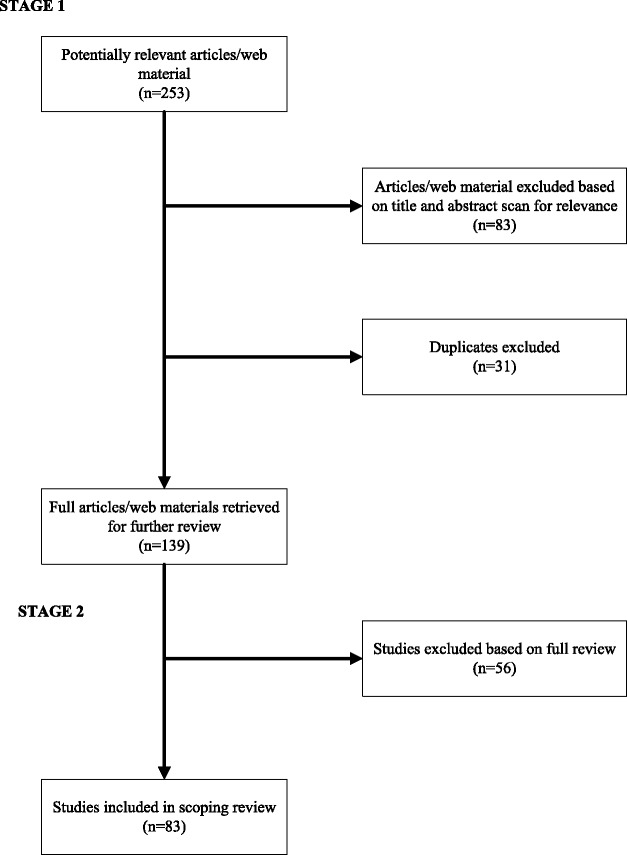
**Illustration of the process of searching and selecting toolkit articles and web material included in the scoping review.**

Source selection was carried out in two stages. Figure 1 illustrates the process of searching and selecting toolkit publications and web-based material. In stage 1, abstracts for all material identified through the search process were located. Two reviewers independently reviewed each title and abstract and consensus was sought on proceeding to review each source in full. Initial exclusion criteria were: 1) toolkits with only one component (e.g., tool, resource); 2) toolkits outside health or education; or 3) toolkits under development. Decision to proceed to full review occurred if: 1) both reviewers agreed to include the abstract; 2) both reviewers found insufficient information to make a determination; 3) both reviewers agreed not to include abstract. Duplicate materials were flagged and conflicts (n = 5) as to whether or not to include an abstract for full review were discussed and resolved. Articles proceeded to full review if they were in the first or second category.

In stage 2 of the scoping review all included and undecided sources were located and reviewed in full. Initial exclusion criteria were again applied and new exclusion criteria emerged; development of exclusion criteria during the review stage is a unique feature of scoping reviews and differentiates scoping reviews from systematic reviews [11]. Given the broad nature of the search it was unlikely that all exclusion criteria could be pre-determined. Sources were further eliminated if they: 1) did not contain the word kit or toolkit, and 2) had insufficient information to make a determination about its inclusion.

Two reviewers independently read all 139 sources identified for full review, with the exception of one article that could not be located. The reviewers periodically conferred on inclusion or exclusion criteria. Conflicting decisions (n = 7) were resolved through reviewer discussion, culminating in the inclusion of 83 sources.

### Data extraction and analysis

Two reviewers independently abstracted the following data from each source: toolkit format, toolkit topic (as specified by the toolkit authors), target audience(s) (e.g., doctors, nurses, etc.), toolkit content, evidence underlying toolkit content, KT goal of the toolkit, whether toolkit had been evaluated, evaluation approach, evaluation design, evaluation outcome. A unique aspect of our review was a focus on whether the toolkit content was evidence-based, which we assessed based on information provided by the authors. Publication details and abstracted data were subsequently transferred to a detailed spreadsheet, and authors met to discuss and synthesize the findings. During meetings, the authors reviewed the information extracted from the sources and organized it into larger categories determined through group consensus discussions.

## Results

### Breadth and format of toolkits used in health and healthcare practice

Of 253 citations initially identified, 139 met initial inclusion criteria and 83 toolkits were included in the final sample. Among the 83 toolkits, the majority (n = 68) were identified in journal articles, and 15 toolkits were located online in their entirety with no related peer-reviewed publication.

Toolkit topics were categorized as clinical, general health and safety, developmental and mental health, technology-related, advocacy, politics, and cultural awareness (see Table 1). Most toolkits (n = 67, 81%) were related to clinical or general health and safety issues (e.g., disease prevention and care, oral health, end of life care). All toolkit topics relating to healthcare practice by healthcare practitioners were considered clinical, as were hospital-specific toolkits with topics related to the practice of patient care in a hospital setting (e.g., infection control, operating room fire prevention, discharge practice, in-patient falls, etc.). Health topics relevant to the general population were categorized as general health and safety (i.e., seat belt use, sun safety).

**Table 1:** **Toolkit topics and audience type**

**Toolkit topic**	**Frequency**
Clinical	41
General health & safety	26
Developmental & mental health	12
Technology	2
Advocacy, politics, cultural awareness	2
**Toolkit audience**	
Healthcare practitioners	70
Community partners	11
Health decision makers/policy makers	10
School	8
Parents and caregivers	5
Other	3

With respect to target audiences, most toolkits (n = 70, 84%) were directed at healthcare practitioners, such as physicians and nurses (see Table 1). Among these, 4 toolkits also targeted patients. Community audiences (e.g., church groups) and health decision makers/policy makers (e.g., organization managers, government officials) were the target of a further 21 (25%) toolkits. School staff, parents and caregivers, and other audiences such as researchers comprised the target for the remaining 16 (19%) toolkits. Many toolkits had more than one target audience, and, consequently, more than one KT goal.

The majority of toolkits (n = 57, 69%) were designed to inform practice change (e.g., changing clinical or organizational practices and procedures, such as needle stick prevention, cancer screening protocols) targeting healthcare practitioners. Educating or sharing knowledge and informing were the main KT goals for 40 (48%) toolkits (e.g., public awareness campaigns such as sun safety) and typically the target audience was parents, caregivers and school staff. Finally, informing policy and decision-making was the KT goal for 10 (12%) toolkits (e.g., Scotland’s national approach to improving mental health services).

Toolkit content was well described in only 36 (43%) of the sources reviewed (see Table 2). All online toolkits fully described toolkit components, while the remaining 57% of sources stated the KT goal and the target audience(s) but did not adequately describe the materials included. Most toolkits included a combination of written materials (e.g., tip sheets, information sheets, guidelines; a toolkit for health professionals and their patients with pre-diabetes [14]), with fewer incorporating audio-visual materials such as CDs and DVDs along with written information (e.g., toolkit for healthcare practitioners to help prevent meningococcal disease [15]). Five toolkits included tools such as a pedometer and Body Mass Index wheel in addition to written materials (e.g., a proper exercise and nutrition toolkit for healthcare practitioners [16]).

**Table 2:** **Toolkit format and content (n = 83 toolkits)**

**Content categories**	**Frequency**
Written materials only	17
Written materials and A.V. material	4
Written materials and other tools/resource	11
Written materials, A.V. materials and other tools/resources	4
Contents not listed	47

### Evidence underlying toolkit content: is the toolkit based on research evidence?

We examined whether the 83 toolkits were evidence-based (i.e., reference or inclusion of evidence underlying the toolkit), and found that 72% included some mention of the evidence supporting the toolkit, while the remaining 28% made no reference to evidence. The type of evidence underlying the development of the 60 toolkits varied widely. Here, evidence took the form of literature scan/reviews (n = 31), qualitative data (i.e., focus groups, interviews or stakeholder surveys; n = 17), expert panels (n = 13), and evidence-based guidelines (n = 14). Toolkits supported by evidence from randomized controlled trials were much less common (n = 3). Toolkits were sometimes supported by multidisciplinary specialists’ discussions, theories or conceptual frameworks, best-practice approaches, reviews of institutional documentation, national reports, and observations of existing practices. Most toolkits relied on a combination of these sources and none of the toolkits specified the evidence base underlying each individual toolkit element; if they were supported by evidence, this was not made clear in the source.

### Effectiveness of toolkits: did the toolkit achieve the intended KT goal(s)?

We also examined whether (1) toolkits as a whole were evaluated relative to their purpose or KT goal, and whether (2) toolkit components were evaluated for specific outcomes. Information regarding evaluation of the toolkit either as a whole or for its components was only available for 31 of the 83 toolkits. Importantly, none of the online toolkits included information about toolkit effectiveness. Table 3 summarizes evaluation type and outcome for the 31 toolkits that were evaluated. Among these, four evaluated the individual toolkit components, while the remaining 27 toolkits were evaluated in their entirety. Toolkit evaluations were typically carried out via interviews and surveys and, more rarely, via focus groups. The most comprehensive evaluation was detailed in *High School Coaches’ Assessments, Intentions to Use and Use of a Concussion Prevention Toolkit* [17], which consisted of an information letter and brochure, reference cards, fact sheets, posters and a video. This was the only toolkit that provided outcome information regarding evaluation of the individual components and reported an almost 100% satisfaction rate.

**Table 3:** **Toolkit evaluation details for the 31 out of 83 toolkits that have been evaluated**

**Evaluation type**	**Frequency**
Process	13
Outcome	8
Process and outcome	8
Insufficient information	2
**Evaluation outcome**	
Toolkit was satisfactory and/or useful	21
Toolkit produced mixed reviews or modest & unsustainable results	2
Toolkit was satisfactory if recommended changes made	4
Less than half of those who responded to the survey used the toolkit	1
Outcome data in process of being collected and analyzed	1

Where possible, information was collected regarding the type of evaluation conducted on the toolkit: process (i.e., evaluation aimed at testing and monitoring the process of toolkit use), and outcome (i.e., evaluation aimed at assessing changes in practice and knowledge as a result of the toolkit use). For instance, the *Toolkit for New Parents* [18], designed for parenting education, has been evaluated both in terms of process (i.e., interviews conducted with state officials, administrators, and mothers to assess the toolkit’s use and customization details) and outcome (i.e., knowledge assessment at baseline and at 2- and 14-months follow-up in mothers who did or did not receive the toolkit).

With respect to overall outcome evaluation, the majority (n = 21) of evaluated toolkits reported that the toolkit was satisfactory, useful, or resulted in an intention to change practice. For instance, 92% of the nurses who received and tried out the *Proper Exercise and Nutrition Toolkit* planned to incorporate it into their practice [16]. Results for two toolkit evaluations showed inconclusive outcomes (i.e., *Safety of the Land Toolkit* designed to increase rural children’s knowledge about safety [19]) and modest initial positive changes that were not sustained at follow-up (i.e., the *Diabetes Literacy and Numeracy Education Toolkit* designed to improve glycemic control and self-efficacy in patients with diabetes [20]). In the first case, there was wide variability in children’s perception and understanding of the message communicated through the safety toolkit components, with some children completely misinterpreting the visual materials provided.

## Discussion

Toolkits as a KT strategy have popular appeal, particularly given the ease with which they can be disseminated on the Internet in an engaging and multimodal manner to a wide variety of audiences. This study set out to scope the literature on toolkits in health and healthcare, to explore their breadth of topic, format, target, content, underlying evidence and their effectiveness as KT strategies. The majority of toolkits focus on clinical and general health and safety topics such as disease prevention and care, oral health, and end of life care. Toolkits permeate both published and grey literature and tend to be targeted to healthcare practitioners, although many also target community-based knowledge users, health decision makers and policy makers. Toolkit content was adequately described in fewer than half of the sources reviewed. Although about 70% of the sources mentioned the evidence underlying the toolkit content, there was a wide range of variability in what constituted this evidence. The most commonly used sources of evidence included literature review findings, qualitative data, expert panels, and practice guidelines. Of the 83 toolkits included in the study, only 31 had been evaluated in any way. Among these, the majority focused on the effectiveness of the toolkit as a whole, and, very rarely, on the effectiveness of the individual components. Among evaluated toolkits, outcomes were generally positive related to their intended KT goal.

To our knowledge, this is the first scoping review on toolkit use in health and healthcare. Our findings indicate that there is sufficient research on this topic to warrant a full systematic review. In addition, the findings reveal several knowledge gaps which can inform how to best share research evidence in a way that optimizes its use. First, evaluation findings suggest toolkits can be an effective KT strategy, but more systematic evaluations are needed to capture impacts beyond intention to use or user satisfaction, which were the most commonly employed indicators of outcome. Systematic evaluations of toolkits that aim to change knowledge, attitudes, practice and, where applicable, patient outcomes, are crucial for understanding their full impact on health practice and behavior, and ultimately strengthen their utility. This finding aligns with results from the Cochrane review on the use of educational materials to change the practice of healthcare professionals and patient outcomes [9]. This gap is particularly relevant for the online toolkits reviewed here, of which none included information about effectiveness.

Second, comparisons of toolkits with other KT strategies using comprehensive designs will help us to elucidate the contexts in which toolkits are useful and the relative advantage of this type of KT strategy. Toolkit research must also include full documentation of the toolkit materials, a limitation noted in 60% of the sources reviewed herein. This is informative for toolkit development, assessing toolkit feasibility and effectiveness, and comparisons to other toolkits and KT educational approaches. Finally, given that toolkits aim to translate knowledge, it is essential to make explicit the type and quality of evidence that is being translated and that underlies each component within the toolkit; in other words, the evidence on which the toolkit elements are based.

We harken back to the conclusion of an earlier review [4] that noted that the effectiveness of KT strategies is highly dependent on context and that no single KT strategy has been shown to be universally effective. In light of this finding, evaluation of whether any KT strategy achieves its knowledge translation goal (e.g., build awareness, share knowledge, impact policy, change behavior or practice) and the subtle contextual factors that facilitate or hinder this outcome, is an essential component of the KT process, and is lacking in our current state of KT practice and science.

We note two limitations. First, we reviewed toolkits developed prior to 2011 and it is likely that other toolkits have been published in the last three years. This presents an opportunity for other authors to extend the review when conducting a more systematic review. Additionally, findings are limited to studies published in English. Despite these limitations, this scoping review presents a comprehensive profile of toolkit use in health and healthcare, and contributes to advancing knowledge in the field of KT. Addressing the above noted gaps in future research has the potential to optimize the effectiveness of toolkits as a strategy to share knowledge and change practice.

## Conclusion

Toolkits are proving to be desirable, accessible, and useful but they lack scientific rigor with respect to the evidence underlying their content, and evaluation of their overall effectiveness. To the extent that toolkits are focused on practice or behavior change, it is imperative that they be supported by research evidence, which was not the case for all toolkits included in this review. Furthermore, fewer than half the toolkits identified as relevant for this review included any evaluation of the toolkit at any level. Moreover, if we are to develop confidence in the ability of toolkits to change practice and/or behavior, we must evaluate and accumulate evidence of their impacts. Only then can we be certain we are behaving ethically, supported by evidence, and with the knowledge that the toolkit is both effective and a defensible investment of time and resources.

## Endnote

^1^References included in the scoping review [13-95].
